# “I BELIEVE I CAN WORK, BUT THEY THOUGHT I CAN’T WORK”: THE WORKING CONDITIONS OF PEOPLE WITH YOUNG ONSET DEMENTIA

**DOI:** 10.1093/geroni/igae098.0664

**Published:** 2024-12-31

**Authors:** Zih-Ling Wang

**Affiliations:** University of Washington, Seattle, Washington, United States

## Abstract

“Young-onset dementia” is a medical condition where a person is diagnosed with dementia before the age of 65. Experiencing behavioral changes and committing mistakes in the workplace due to dementia can lead to issues with employers, pension funds, and financial matters. However, there have been few studies on individuals with young-onset dementia or their working conditions in Taiwan. A qualitative research design was utilized to conduct semi-structured in-depth interviews from three perspectives: people with young-onset dementia, family care partners, and employment specialists. This approach aimed to gain insights into whether and how reasonable accommodation could be implemented in the workplace before COVID-19, how affected individuals adapt to work, and how family care partners and employment specialists assist them in continuing their employment. The research identified three main themes: (1) adaptive strategies and resilience in the workplace among young onset dementia, (2) advocating for supportive environments, and (3) empowering individuals with young onset dementia through tailored career support. This study highlighted areas for improvement in protecting the right to work and ensuring reasonable accommodation for young onset dementia, emphasizing the need for appropriate adjustments in their work environment. The goal is for the public to recognize the capabilities of such individuals and to safeguard their right to work.

